# Editorial: Proteostasis in cardiac health and disease

**DOI:** 10.3389/fmolb.2024.1433721

**Published:** 2024-05-31

**Authors:** Hannah Bühringer, Shirin Doroudgar, Xuejun Wang, Norbert Frey, Ashraf Yusuf Rangrez

**Affiliations:** ^1^ Department of Cardiology, Angiology and Pneumology, University Hospital Heidelberg, Heidelberg, Germany; ^2^ DZHK (German Centre for Cardiovascular Research), Partner Site Heidelberg/Mannheim, Heidelberg/Mannheim, Germany; ^3^ Department of Internal Medicine and the Translational Cardiovascular Research Center, University of Arizona College of Medicine–Phoenix, Phoenix, AZ, United States; ^4^ Division of Basic Biomedical Sciences, University of South Dakota Sanford School of Medicine, Vermillion, SD, United States

**Keywords:** cardiac proteostasis, desminopathies, protein agglomeration, ubiquitin proteasomal system, heart failure

Cardiovascular diseases (CVDs) are the leading cause of death worldwide. The past few decades saw witness to exhilarating efforts from researchers across the globe to uncover the underlying molecular mechanisms of CVDs. Only recently, however, has protein homeostasis or proteostasis as a key cellular process caught attention in the context of heart failure and cardiomyopathy (Wang and Robbins, 2006). Proteostasis involves complex and tightly regulated processes to balance the production, folding, trafficking, and degradation of cellular proteins (Henning and Brundel, 2017). Protein synthesis and folding are monitored by a specialized system, collectively called Protein Quality Control (PQC), where chaperones, the Ubiquitin-Proteasome System (UPS), and sometimes autophagy act as central players. PQC impairment or inadequacy may result in aberrant protein aggregation which in turn impairs the UPS and further increases proteotoxic stress, which may eventually lead to cell death (Wang and Wang, 2020). Due to the exceptionally high mechanical, thermal, and oxidative stresses in working cardiomyocytes, maintaining their proteostasis is particularly challenging but crucial for the health and function of these cells and the heart (Henning and Brundel, 2017). Accumulation of misfolded proteins and aberrant protein aggregation are now even considered a hallmark of a large subset of cardiac diseases (Sanbe et al., 2004; Hofmann et al., 2019; Wang and Wang, 2020). However, we are still far from understanding the exact mechanisms regulating cardiac proteostasis.

The aim of the articles in this Research Topic is to shed light on the still undiscovered aspects of proteostasis in the heart and its role in cardiac health and disease. Of particular interest hereby is the UPS. UPS-mediated proteolysis is the primary degradation system to remove aberrant proteins. Alterations of the UPS have been reported in several human and experimental cardiac disorders (Mearini et al., 2008; Schlossarek et al., 2014). Consistent with this, Trogisch et al. investigate whether lack of LMP2, an immune or inducible subunit of the 20S proteasome, and thereby reduced proteasome plasticity, impact cardiac remodeling and function under chronic β-adrenergic stimulation, a major contributor to the development of heart failure. While cardiac function of LMP2 constitutive knock-out (KO) mice remained unchanged under unchallenged conditions, continuous β-adrenergic stimulation led to augmented cardiac hypertrophy, uncoupling of isoproterenol induced cardiac function, and cardiac contractile deficit. Unexpectedly, there was no evidence of intracellular misfolded protein accumulation. Instead, the authors found a significant increase of extracellular matrix proteins collagen I and III, causing an impaired diastolic function in LMP2 KO mice. On the one hand, these results show that therapeutic targeting of LMP2 should be avoided due to the subsequent deterioration in cardiac function. At the same time, the question is raised as to whether the positive role of LMP2 in the context of cardiology can be therapeutically exploited.

Another important component of the UPS is deubiquitinating enzymes (DUBs). DUBs catalyze the removal of ubiquitin from ubiquitinated proteins to prevent degradation by the proteasome. The review by Wang et al. illustrates the role of ubiquitin-specific protease (USP)-13 in the regulation of different cellular mechanisms and its contribution to several disease conditions, providing new insights into the prevention and treatment of relevant diseases, such as neurodegenerative diseases or cancer. So far, very little is known about the role of USP-13 in the heart. However, the detailed insights provided in this review and the results of a recent study by Wu et al. give rise to the possibility of USP-13 involvement in cardiac diseases (Wu et al., 2023).

Recent evidence suggests that the cytoskeleton of cardiomyocytes is crucial for maintaining balanced communication between the various components of the proteostasis network (Henning and Brundel, 2017). One of the critical components of the cytoskeleton is the intermediate filament protein, desmin. Genetic variations and loss of desmin cause desminopathies, including desmin-related cardiomyopathies. The review by Su et al. discusses the role of desmin impairment as a trigger for cardiac arrythmias and the underlying molecular mechanisms.

A derailment of PQC with the formation of aberrant protein aggregates can have drastic effects in cardiac diseases (Wang and Robbins, 2006; Henning and Brundel, 2017). Petersen et al. discovered Fibin, fin bud initiation factor homolog, as a novel protein induced in cardiac hypertrophy*. In vitro*, using neonatal rat ventricular cardiomyocytes, they found Fibin to act as a regulator of cardiomyocyte hypertrophy through inhibition of SRF and NFAT signaling, two well-described pathways involved in cardiomyocyte hypertrophy. In the expectation of a positive effect on cardiac hypertrophy, they next investigated the role of Fibin *in vivo*, using a cardiomyocyte-restricted Fibin overexpression mouse model. Surprisingly however, these transgenic mice developed dilated cardiomyopathy and cardiac dysfunction in aging, pressure-overload, and calcineurin mouse models. The cause of this unexpected phenotype was identified as the result of a proteostasis perturbation leading to aberrant protein aggregation, increased ER stress, and the Uncoupled Protein Response (UPR), resulting in UPR-mediated apoptosis. Thus, this study demonstrates the importance of balanced proteostasis and exemplifies the potentially devastating effects of dysregulated proteostasis on the heart.

Overall, these studies demonstrate the importance and complexity of cardiac proteostasis. While research into cardiac proteostasis is still in its infancy, protein homeostasis has been implicated in the context of aging. As we age, proteolytic activity declines, resulting in the accumulation of damaged proteins and aggregates, which are causal in the onset of several chronic diseases (Kaushik and Cuervo, 2015). Best characterized are the effects of toxic accumulations in the age-related neurodegenerative diseases: Parkinson’s, Alzheimer’s, and Huntington’s Disease (Taylor and Dillin, 2011). However, more and more evidence suggests that a decline in PQC may be causal for the development of other widespread diseases, including obesity and type 2 diabetes (Taylor and Dillin, 2011; Kaushik and Cuervo, 2015; Guerra et al., 2024). The health burden caused by the aforementioned diseases requires multidisciplinary studies to elucidate a possible link between the derailment of proteostasis in different diseases. One intriguing possibility is the idea of treating multiple diseases by therapeutically improving proteasome function (Wang and Wang, 2020). One potential and relatively recent approach is the activation of the proteasome by small molecules (George and Tepe, 2021). Even though several promising possible targets and compounds are emerging, the consequences and side effects of chronic exposure to proteasome enhancers are not yet known. However, in the future, therapeutically targeting proteostasis could open completely new possibilities in the treatment of widespread diseases, including CVDs.
